# Integral Health Status-Based Cluster Analysis in Moderate–Severe COPD Patients Identifies Three Clinical Phenotypes: Relevant for Treatment As Usual and Pulmonary Rehabilitation

**DOI:** 10.1007/s12529-016-9622-3

**Published:** 2016-12-19

**Authors:** Jeannette B. Peters, Lonneke M. Boer, Johan Molema, Yvonne F. Heijdra, Judith B. Prins, Jan H. Vercoulen

**Affiliations:** 1 Department of Pulmonary Diseases, Radboud University Medical Center, Nijmegen, The Netherlands; 2 Department of Medical Psychology, Radboud University Medical Center, Postbus 66, 6560 AB Groesbeek, Nijmegen, The Netherlands

**Keywords:** Integral health status, Clinical phenotypes, Adaptation, Pulmonary rehabilitation, Cluster analysis, COPD

## Abstract

**Purpose:**

The purposes of the study are to identify clinical phenotypes that reflect the level of adaptation to the disease and to examine whether these clinical phenotypes respond differently to treatment as usual (TAU) and pulmonary rehabilitation (PR), the latter with its strong emphasis on improving adaptation.

**Methods:**

Clusters were identified by a cluster analysis using data on many subdomains of the four domains of health status (HS) (physiological functioning, functional impairment, symptoms and quality of life) in 160 outpatients with chronic obstructive pulmonary disease (COPD) receiving TAU. By discriminant analysis in the TAU sample, all 459 PR patients could be assigned to one of the identified clusters. The effect of TAU and PR on HS was examined with paired *t* tests.

**Results:**

Three distinct phenotypes were identified in the TAU sample. Two types were labelled adapted: phenotype 1 (moderate COPD–low impact on HS, *n* = 53) and phenotype 3 (severe COPD–moderate impact on HS, *n* = 73). One type was labelled non-adapted: phenotype 2 (moderate COPD–high impact on HS, *n* = 34). After 1-year TAU, the integral health status of all patients did not improve in any subdomain. In contrast, at the end of PR, significant improvements in HS were found in all three phenotypes especially the non-adapted.

**Conclusions:**

Different phenotypes exist in COPD that are based on behavioural aspects (i.e. the level of adaptation to the disease). Non-adapted patient responds better to treatments with a strong emphasis on improving adaptation by learning the patient better self-management skills. Knowing to which clinical phenotype a patient belongs helps to optimize patient-tailored treatment.

## Introduction

‘Chronic obstructive pulmonary disease (COPD) is the fourth leading cause of death in the world and is a common, preventable and treatable disease characterized by persistent airflow limitation that is usually progressive and associated with enhanced chronic inflammatory response in the airways and the lung to noxious particles or gases’ [1]. The various pulmonary and extrapulmonary manifestations of COPD make it a complex and heterogeneous disorder [2]. In the past years, the acknowledgement of the heterogeneity of COPD has led to an increasing number of studies attempting to identify homogeneous subgroups. The hypothesis of these studies is that each subgroup responds differently to (pharmacological) treatment and has a different course in time. Identification of phenotypes will enhance patient-tailored treatment and improve outcome [3–5].

To date, most studies focused on identification of phenotypes based mainly, and sometimes exclusively, on the (patho)physiological disease characteristics of COPD [6–10]. However, a patient with COPD not only experiences physiological disturbances but also has symptoms, functional impairments and a lowered quality of life. The domains symptoms, functional impairments and quality of life are poorly related to (patho)physiological aspects and the physiological functioning [2, 11–13]. This can be observed in clinical practice where some patients report more severe symptoms, functional impairments or lower quality of life than is expected based on physiological test results, and vice versa.

Symptoms, functional impairments and quality of life are determined not solely by physiological functioning but also by the degree to which the patient succeeds to adapt to the illness through adequate self-management behaviours [14–16]. Examples of self-management strategies are adherence to medication regimes, exacerbation management, adopting a healthy lifestyle (stop smoking, regular exercising), energy-saving strategies, breathing regulation and stress management. Adopting self-management strategies by the patient will result in better adaptation to the disease, and subsequently, the patient will experience less impact on health status (HS). Note that adequate adaptation requires behaviour change by the patient [14, 16]. Not all patients succeed to change behaviour and as a result may suffer from more severe symptoms, functional impairments and lower quality of life than would be expected based on physiological functioning. Identification of clinical phenotypes reflecting the degree of adaptation to the disease could be of added value in addition to the (patho)physiological phenotypes in guiding patient-tailored treatment.

The primary aim of the present study was to investigate whether clinical phenotypes can be identified that reflect the level of adaptation to the disease using cluster analysis based on detailed assessment of all four domains of integral health status. We hypothesized that adaptation to the disease is reflected by the relative balance between disease severity (i.e. physiological functioning) on one hand and the severity of symptoms, functional impairments and reduced quality of life on the other. In patients who are adapted to the four domains of health status will be in balance, whereas patients who are not adapted to these four domains are not in balance. Although such different profiles can be observed in clinical practice, such profiles including all four domains of health status have not yet been identified through empirical studies. Burgel et al. [4] already found that their identified clinical phenotypes were not based on airflow limitation and showed marked differences in quality of life and symptoms but did not include the role of functional impairment. The secondary aim of the study was to explore if these clinical phenotypes respond differently to treatment as usual (TAU) and to a multidisciplinary pulmonary rehabilitation program (PR), which includes an intensive array of interventions aimed at improving adaptation to the disease by teaching the patient adequate self-management strategies in addition to exercise training.

## Methods

We used two different datasets in the present study. For the identification of the clinical phenotypes by cluster analysis, we used a dataset of 160 stable outpatients with COPD receiving TAU at an outpatient clinic. We expected that in this sample, groups of patients could be identified: those who are adapted to the disease and those patients who are not. In this sample, we also investigated the course in time over a 1-year period.

To investigate response to treatment, of the identified phenotypes, we used a sample of patients enrolled in an inpatient multidisciplinary pulmonary rehabilitation program (PR group). This pulmonary rehabilitation program aims at improving integral health status (i.e. physiological functioning, symptoms, functional impairments and quality of life) and contains not only interventions to improve physiological functioning (e.g. exercise training) but also a wide array of interventions to improve the adaptation to the disease by teaching the patient self-management behaviours (e.g. education and specific cognitive behavioural interventions). This program is in line with the recent ATS/ERS statement on pulmonary rehabilitation [17]. We expected that the majority of the patients in this sample could be labelled as non-adapted at the start of the program and would benefit more from the pulmonary rehabilitation program as compared to the group of adapted patients.

### Participants

#### Treatment As Usual (TAU Group)

Outpatients with stable COPD were recruited between 2002 and 2005 as part of a longitudinal study on integral health status in COPD [12] at the University Lung Centre Dekkerswald of the Radboud University Medical Center, Maas Hospital Boxmeer and Rijnstate Hospital Arnhem, the Netherlands. During 1 year, all patient charts were screened by a pulmonologist, which resulted in 361 eligible patients, of whom 168 (47%) eventually participated. Complete datasets at baseline were present of 160 outpatients. COPD was diagnosed by the presence of a post-bronchodilator forced expiratory volume in 1 s (FEV_1_) to forced vital capacity (FVC) ratio of <70% according to the Global Initiative for Chronic Obstructive Lung Disease (GOLD) guidelines [1, 18]. Only patients with an FEV_1_% of predicted between 30 and 80% (GOLD grades 2–3) were included. Exclusion criteria were comorbidity dominating integral health status, an acute exacerbation, participation in pulmonary rehabilitation program (within 6 months) and inability to completely adhere to the research protocol. A detailed description of the recruitment procedure, the study sample and measurements can be found elsewhere [11]. The study was approved by the local ethics committee (P02.1411L; CMO-nr2002/047), and informed consent was obtained from all participants.

#### Pulmonary Rehabilitation (PR Group)

Complete datasets were collected of 459 patients with COPD who completed a 12-week (5 days a week) inpatient multidisciplinary pulmonary rehabilitation program at the University Lung Centre Dekkerswald of the Radboud University Medical Center, between July 2002 and July 2013 as part of usual care. Based on extensive assessments and clinical interviews by seven disciplines (pulmonologist, psychologist, physiotherapist, nurse, dietician, psychomotor therapist, social worker) goals were set for the pulmonary rehabilitation program for each individual patient. During 12 weeks, the patients followed a multidisciplinary and individualized treatment program, consisting of a training program, education sessions, group therapy and individual therapy. Every 3 weeks, the treatment progress was evaluated by the seven disciplines and with the patient. If necessary, the treatment program was adapted. Exclusion criteria for the present study were inability to speak or read Dutch and/or an incomplete dataset. Data collection was part of usual care and anonymized before analyses.

### Procedure

Baseline assessments for the samples were performed during 2 days in the TAU group and during 3 days in the PR group. During the first visit, pulmonary function tests, bioelectrical impedance and maximal incremental cycle ergometry testing were performed. During the second visit, data were collected on demographics, tobacco smoking and self-reported comorbidities. Integral health status was measured by the Nijmegen Clinical Screening Instrument (NCSI) [19]. The NCSI is a battery of existing instruments that was empirically composed such that overlap between instruments is avoided and that a wide variety of aspects of integral health status is measured. The NCSI measures 11 subdomains of integral health status (see Table 1) [11, 20–24]. A higher score on a subdomain means more problematic.

**Table 1:** Health status subdomains and their definition and included instruments of the Nijmegen Clinical Screening Instrument (NCSI)

Domain	Subdomain	Definition	Instruments/measurement	No. of items
Physiological functioning	Airflow		Post-bronchodilator FEV_1_% of predicted	
	Body composition		Body mass index	
	Exercise capacity		VO_2_ max% of predicted	
Symptoms	Subjective symptoms	The patient’s overall burden of pulmonary symptoms	PARS-D Global Dyspnoea Activity [11]	1
			PARS-D Global Dyspnoea Burden [11]	1
	Dyspnoea emotions	The level of frustration and anxiety a person experiences when dyspnoeic	DEQ frustration [11]	3
			DEQ anxiety [11]	
	Fatigue	The level of experienced fatigue	CIS subjective fatigue [20]	8
Functional impairment	Subjective impairment	The experienced degree of impairment in general	QoLRiQ general activities [21]	4
	Behavioural impairment	The extent to which a person cannot perform specific and concrete activities as a result of having the disease	SIP home management [22]	10
			SIP ambulation [22]	12
Quality of life	General quality of life	Mood and the satisfaction of a person with his/her life as a whole	BDI primary care [23]	7
			Satisfaction with life scale [24]	5
	Health-related quality of life	Satisfaction related to physical functioning and the future	Satisfaction physiological functioning [11]	1
			Satisfaction future [11]	1
	Satisfaction relations	Satisfaction with the (absent) relationships with spouse and others	Satisfaction spouse [11]	1
			Satisfaction social [11]	1

*No*. number, *FEV*_*1*_ forced expiratory volume in 1 s, *VO*_*2*_ *max* maximal oxygen uptake, *PARS-D* Physical Activity Rating Scale-Dyspnoea, *DEQ* Dyspnoea Emotions Questionnaire, *CIS* Checklist Individual Strength, *QoL-RiQ* Quality of Life for Respiratory Illness Questionnaire, *SIP* Sickness Impact Profile, *BDI* Beck’s Depression Inventory

In the PR sample, on the second and third day, interviews by seven disciplines took place.

All assessments (except incremental cycle ergometry) were repeated after 1 year in the TAU group (complete datasets of 143 patients) and at the end of the rehabilitation in PR group (complete datasets of 459 patients).

### Statistical Methods

All analyses were conducted using IBM SPSS statistics version 20 (SPSS Inc., Chicago, IL).

Firstly, to identify clinical phenotypes in the TAU sample based on integral health status profiles (primary aim), we conducted a hierarchical cluster analysis using Ward’s method (with squared Euclidean distance) [4, 25]. Ward’s cluster analysis is applied when there is no prior knowledge about the number of clusters or how the clusters may be characterized. In this analysis, grouping is based such that subjects in the same cluster are more similar to each other than to subjects in other clusters. To form clusters, we included the following 11 parameters: FEV_1_% of predicted, body composition (BMI kg m^−2^), exercise capacity (VO_2_ max% of predicted), subjective symptoms, dyspnoea emotions, fatigue, subjective impairment, behavioural impairment, general quality of life, health-related quality of life and satisfaction with relationships. Based on the dendogram, the optimal number of clusters was identified.

Second, we performed a one-way ANOVA and Tukey’s post hoc test to determine whether the clusters significantly differ from each other on the included variables.

Third, a (stepwise) discriminant function analysis was performed on the TAU sample to determine which parameters were most discriminatory between the clusters. The discriminant cluster analysis created an equation, which allows assigning new cases to the identified clusters. This equation was used to assign each of the 459 PR patients into a cluster.

To examine change in integral health status over time in TAU and PR, several analyses were performed (secondary aim). To analyse change over time in integral health status subdomains in TAU, paired *t* tests were performed for each subdomain in outpatients who completed the assessment at baseline and 1 year later (*N* = 143, 89.4%). For each subdomain, the score at baseline was compared to the score after 1 year (except for exercise capacity, because the maximal ergometry test was not performed after 1 year). These analyses were performed on the whole TAU group and for each cluster separately.

To examine response to treatment (i.e. improvement in integral health status), paired *t* tests were performed in 459 patients of the PR group. The scores on the 11 outcome measures before rehabilitation were compared to the scores at the end of rehabilitation for the whole PR group and for each of the clusters separately.

All statistics are presented as mean ± standard deviation (SD) or percentage (number of patients, *n*). Differences between clusters on sex, GOLD grade, nutritional status, tobacco use and education were tested with Pearson’s chi-squared test, and differences between clusters on age, FEV_1_/FVC ratio and number of self-reported comorbidities were analysed with one-way ANOVA. *Z* scores were calculated to allow for comparisons of the different subdomains and to illustrate the relative distance from the total group mean (*Z* score = 0). *Z* scores were based on baseline and calculated as (score − baseline mean score of the TAU group) / baseline standard deviation of the TAU group. Differences in TAU and PR were tested with paired *t* tests. To avoid type I error due to multiple testing, *p* was set at 0.01.

## Results

### Treatment As Usual (TAU Group)

The baseline characteristics of the TAU group are presented in Table 2. Most patients were male, overweight, former smoker, low educated and 72% reported having one or more comorbidities. Due to normal variation in FEV_1_, some patients were classified as GOLD grade 1 or GOLD grade 4 (8%).

**Table 2:** Baseline characteristics of outpatients (TAU group, *N* = 160), for total group, for each identified phenotype and for patients enrolled in the pulmonary rehabilitation program (PR group, *N* = 459)

	Outpatients (*N* = 160)					Pulmonary rehabilitation
	Total group	Phenotype 1 (*n* = 53)	Phenotype 2 (*n* = 34)	Phenotype 3 (*n* = 73)	*p*	
Male	77 (123)	77.4 (41)	70.6 (24)	79.5 (58)	0.06	53.5 (243)
Age	64.2 ± 9.1	65.1 ± 9.3	64.5 ± 8.4	63.4 ± 9.2	0.56	60.5 ± 8.8
FEV_1_/FVC ratio	42.9 ± 11.4	46.9 ± 9.2	46.2 ± 13.0	38.6 ± 10.6		36.7 ± 12.1
GOLD grade					<0.01	
Grade 1 (mild)	2.5 (4)	5.7 (3)	3.0 (1)	–		3.8 (17)
Grade 2 (moderate)	50.3 (80)	73.6 (39)	51.5 (17)	32.9 (24)		22.3 (100)
Grade 3 (severe)	42.1 (67)	20.8 (11)	42.4 (14)	57.5 (42)		47.0 (211)
Grade 4 (very severe)	5.0 (8)	–	3.0 (1)	9.6 (7)		26.9 (121)
Nutritional status					<0.01	
Underweight (BMI <21)	11.9 (19)	7.5 (4)	14.7 (5)	13.7 (10)		14.7 (67)
Normal weight (>21 BMI <25)	35.0 (56)	54.7 (29)	11.8 (17)	31.5 (23)		34.6 (158)
Overweight (>25 BMI <30)	37.5 (60)	30.2 (16)	50.0 (17)	37.0 (27)		32.2 (147)
Obese (BMI >30)	15.6 (25)	7.5 (4)	23.5 (8)	17.8 (13)		18.6 (85)
Tobacco use					<0.01	
Smoker	26.3 (42)	22.6 (12)	20.6 (7)	31.5 (23)		10.8 (49)
Former smoker	60.0 (96)	50.9 (27)	61.8 (21)	65.8 (48)		84.6 (384)
Never smoked	13.8 (22)	26.4 (14)	17.6 (6)	2.7 (2)		4.6 (21)
Education					0.63	
Low	51.6 (82)	50.0 (26)	53.0 (18)	52.0 (38)		51.9 (235)
Middle	30.2 (48)	28.58 (15)	23.5 (8)	34.3 (25)		34.6 (157)
High	18.2 (29)	21.1 (11)	23.5 (8)	13.7 (10)		13.5 (61)
Self-reported comorbidities	1.34 ± 1.25	1.25 ± 1.00	2.41 ± 1.46	0.92 ± 1.02	<0.01	1.53 ± 1.27
None	28.1 (45)	24.5 (13)	5.9 (2)	41.1 (30)		24.0 (109)
Fatigue	30.0 (48)	22.6 (12)	70.6 (24)	16.4 (12)		44.1 (200)
Back pain	30.6 (49)	32.1 (17)	38.2 (13)	26.0 (19)		24.4 (111)
Rheumatoid arthritis	24.4 (39)	15.1 (8)	61.8 (21)	13.7 (10)		20.9 (95)
Psychological problems	6.3 (10)	–	26.5 (9)	1 (1)		16.3 (74)
Diabetes mellitus	5.0 (8)	7.5 (4)	2.9 (1)	4.1 (3)		9.9 (45)
Cancer	3.1 (5)	3.8 (2)	2.9 (1)	2.7 (2)		1.3 (6)
Cardiac disease	7.5 (12)	5.7 (3)	14.7 (5)	5.5 (4)		15.9 (72)
Other	27.5 (44)	37.7 (20)	23.5 (8)	21.9 (16)		20.3 (92)

Data are expressed as % (*N*) or mean ± SD

*FEV*_*1*_ *%* forced expiratory volume in 1 s, *FVC* forced vital capacity, *GOLD* Global Initiative for Chronic Obstructive Lung Disease, *BMI* body mass index

### Identification of Clinical Phenotypes in the TAU Group

**Fig. 1:**
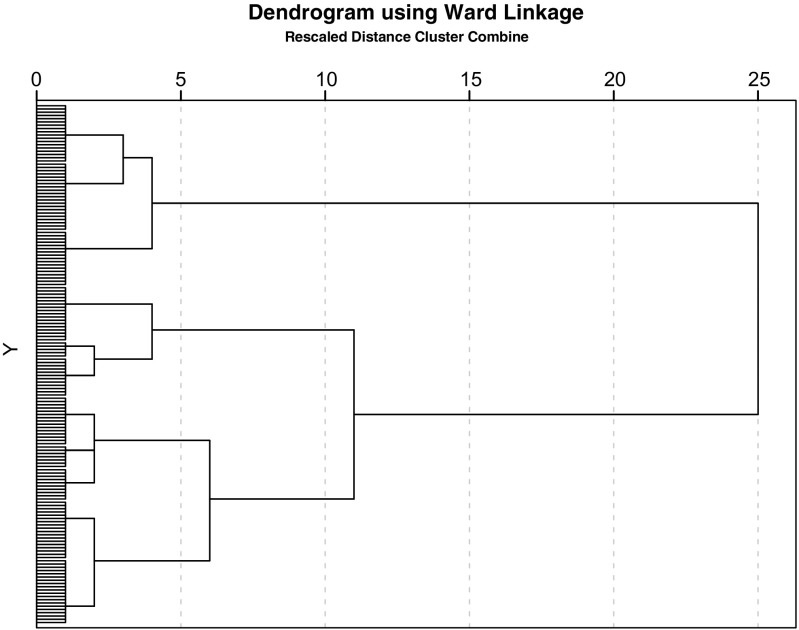
Dendrogram of the identified three distinct clusters based on the hierarchical cluster analysis using Ward’s method

We identified three distinct clusters based on the hierarchical cluster analysis using Ward’s method (Fig. 1). Significant differences were found on baseline characteristics between the three clusters on BMI categories, tobacco use and number of self-reported comorbidities (Table 2). The one-way ANOVA and Tukey’s post hoc test showed that the three clusters were significantly different on all included subdomains, except for body composition (BMI, Table 3).

**Table 3:** Baseline characteristics of 160 outpatients on subdomains of health status, for total group and each phenotype identified by Ward’s cluster analysis

	Outpatients (*N* = 160)		Phenotype 1 (*n* = 53)	Phenotype 2 (*n* = 34)	Phenotype 3 (*n* = 73)	One-way ANOVA	Tukey’s post hoc tests		
Domain/subdomain	Mean ± SD	95% CI	Mean ± SD	Mean ± SD	Mean ± SD		1 vs 2	1 vs 3	2 vs 3
Physiological functioning									
FEV_1_% of predicted	51.7 ± 13.7	49.6 to 53.9	59.8 ± 11.8	53.4 ± 12.0	44.5 ± 12.0	**<0.01**	0.04	**<0.01**	**<0.01**
BMI	25.8 ± 4.0	25.1 to 26.4	24.9 ± 3.0	26.6 ± 4.5	26.0 ± 4.4	0.09	–	–	–
VO_2_ max% of predicted	71.9 ± 19.8	68.9 to 74.8	91.4 ± 13.9	65.8 ± 12.6	59.2 ± 10.0	**<0.01**	**<0.01**	**<0.01**	0.03
Symptoms									
Subjective symptoms	7.2 ± 4.7	6.5 to 7.9	4.5 ± 3.5	10.9 ± 3.8	7.6 ± 4.5	**<0.01**	**<0.01**	**<0.01**	**<0.01**
Dyspnoea emotions	9.8 ± 3.2	9.3 to 10.3	9.3 ± 3.0	11.9 ± 3.6	9.1 ± 2.7	**<0.01**	**<0.01**	0.87	**<0.01**
Fatigue	27.0 ± 11.2	25.2 to 28.7	21.2 ± 7.7	39.4 ± 8.5	25.5 ± 9.7	**<0.01**	**<0.01**	0.02	**<0.01**
Functional impairment									
Subjective impairment	9.8 ± 4.9	9.0 to 10.5	6.9 ± 2.7	15.3 ± 5.0	9.4 ± 3.8	**<0.01**	**<0.01**	**<0.01**	**<0.01**
Behavioural impairment	15.1 ± 13.0	13.1 to 17.2	7.0 ± 6.8	30.0 ± 12.2	14.4 ± 11.0	**<0.01**	**<0.01**	**<0.01**	**<0.01**
Quality of life									
General QoL	14.1 ± 12.1	12.2 to 16.0	9.1 ± 5.7	29.4 ± 14.4	10.6 ± 8.0	**<0.01**	**<0.01**	0.65	**<0.01**
HrQoL	4.1 ± 1.7	3.8 to 4.3	3.2 ± 0.9	5.9 ± 1.6	3.9 ± 1.5	**<0.01**	**<0.01**	0.02	**<0.01**
Satisfaction with relations	3.1 ± 1.4	2.9 to 3.3	2.8 ± 1.1	4.3 ± 1.9	2.8 ± 1.0	**<0.01**	**<0.01**	0.98	**<0.01**

Data are expressed as mean ± SD, *p* values in bold: significant differences between the phenotypes

*FEV*_*1*_ *%* forced expiratory volume in 1 s, *BMI* body mass index, *QoL* quality of life

**Fig. 2:**
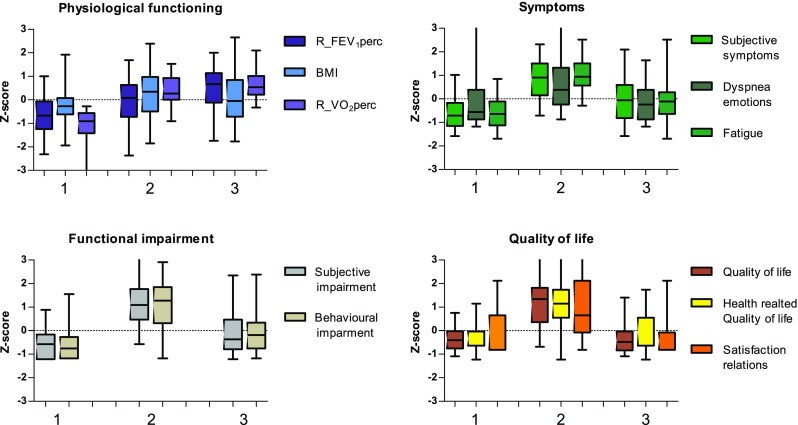
Graphs showing the relationship between phenotype and parameters physiological functioning, symptoms, functional impairment and quality of life

The first cluster (phenotype 1) was characterized by having moderate COPD, normal weight, high performance on exercise capacity and mild impact on symptoms, functional impairments and quality of life (Table 3 and Fig. 2). Cluster 2 (phenotype 2) patients were characterized by moderate COPD, overweight, moderate performance on exercise capacity and with high impact on symptoms, impairment and quality of life. Cluster 3 (phenotype 3) patients were characterized by severe COPD, overweight, moderate performance on exercise capacity and mild (to moderate impact) on symptoms, impairment and quality of life.

Although comparable on FEV_1_% predicted and BMI with phenotype 1, phenotype 2 patients had significantly higher scores on all subdomains of symptoms, functional impairment and quality of life compared to phenotype 1 (Table 3 and Fig. 2). Phenotype 3 patients had significantly poorer FEV_1_% predicted than phenotype 2 but reported a significantly lower impact on symptoms, functional impairment and quality of life when compared to phenotype 2. Remarkably, phenotype 3 had more severe COPD than phenotype 1 but had similar mild impairment in five out of six subdomains of symptoms and quality of life (*p* > 0.01).

Discriminant analysis showed that 95% of the TAU group could correctly be classified by the following five variables: VO_2_% predicted, FEV_1_% predicted, general quality of life, behavioural impairments and fatigue.

### Distribution of the Clinical Phenotypes in the PR Group

Baseline characteristics of the 459 COPD patients of the PR group are presented in Table 2. Almost half was female; most had moderate to very severe COPD, were former smoker and low educated, and 76% reported having one or more comorbidities. Based on the equation from the discriminant analysis (see Appendix Table 4), the patients of the PR group were assigned into one of the three phenotypes. Whereas patients of the TAU group primarily were identified as phenotype 1 ‘moderate COPD with mild impact on integral HS’ or phenotype 3 ‘severe COPD with mild impact on integral HS’ (33 and 46%, respectively), patients in the PR group primarily were identified as phenotype 2 ‘moderate COPD with high impact on integral HS’ (59 vs 21% in the outpatient sample) and only 6% was identified as phenotype 1 moderate COPD with mild impact on integral HS.

### Differences in Change in Integral HS Between Phenotypes in TAU and PR

**Fig. 3:**
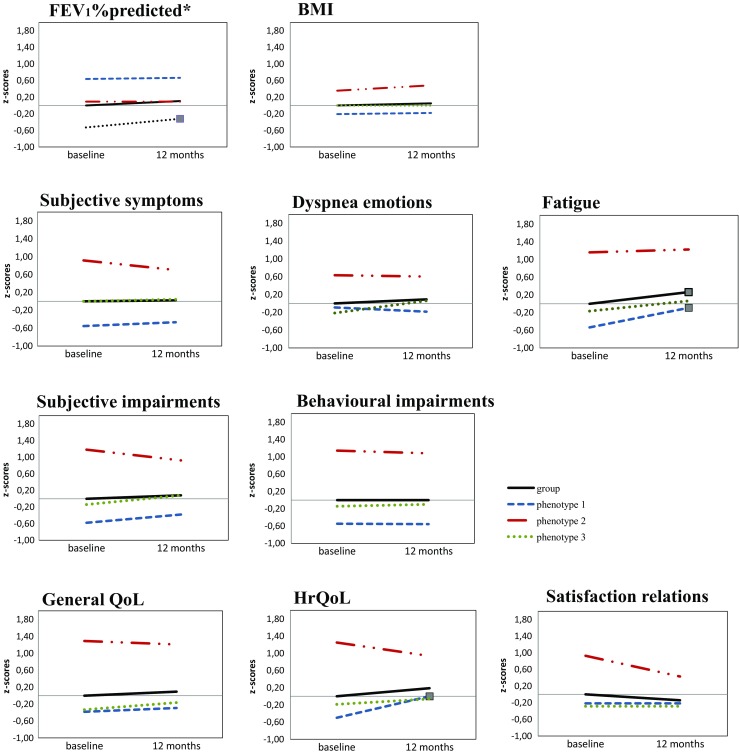
Graphs showing *Z* scores at baseline and 12 months of parameters FEV_1_% predicted, BMI, subjective symptoms, dyspnoea emotions, fatique, subjective impairments, behavioural impairments, general QoL, HrQoL and satisfaction relations

Of the 160 TAU group, 143 patients (89.4%) also participated 1 year later. Reasons for drop out were diverse, but no significant differences were found on the 11 outcome variables nor on the baseline characteristics between the responders and non-responders (data not shown). On a group level, only significant change was found on fatigue between baseline and follow-up (*p* < 0.01, Fig. 3 and Appendix Table 5). Between phenotypes, only very few significant differences were found over the 1-year period in usual care. Phenotype 1 patients had significantly higher scores (more problems) in fatigue and health-related quality of life, and phenotype 3 patients had significantly better FEV_1_% predicted (*p* < 0.01, Fig. 3 and Appendix Table 5) after 12 months.

**Fig. 4:**
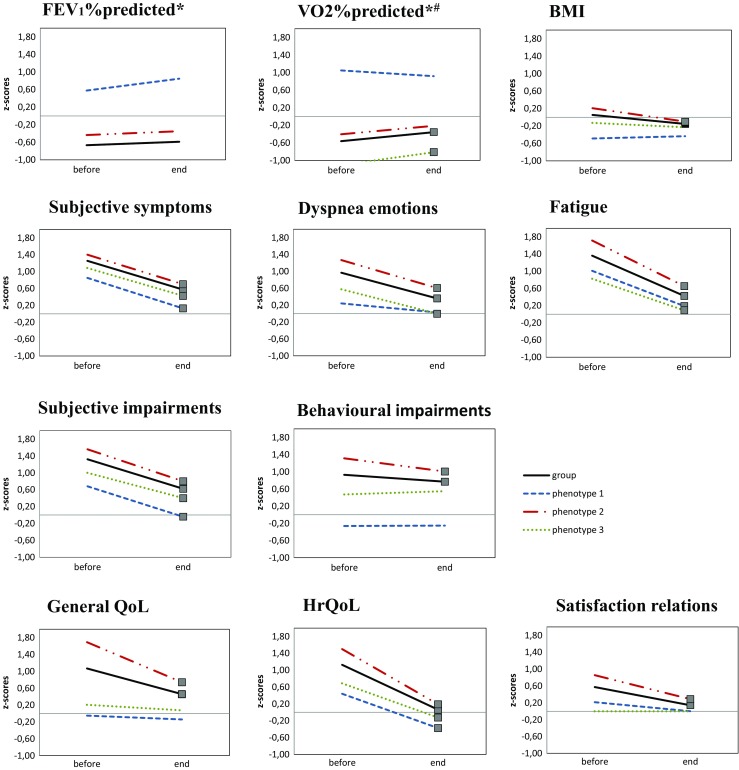
Graphs showing Z scores before and end of pulmonary rehabilitation of parameters FEV_1_% predicted, VO_2_% predicted, BMI, subjective symptoms, dyspnoea emotions, fatique, subjective impairments, behavioural impairments, general QoL, HrQoL and satisfaction relations

Significant improvements were found in 10 of 11 subdomains of symptoms, functional impairment and quality of life in the total PR group when post-rehabilitation scores were compared to pre-rehabilitation scores (*p* < 0.01, Fig. 4 and Appendix Table 6). Major differences were found between the three phenotypes in the number of significantly improved subdomains at the end of rehabilitation, varying from 4 (phenotype 1) to 10 significantly improved subdomains (phenotype 2) (Fig. 4 and Appendix Table 6).

## Discussion

### Main Findings

In the present study, we empirically identified three clinical phenotypes using cluster analysis based on a wide variety of parameters measuring aspects of physiological functioning, symptoms, functional impairment and quality of life in a group of outpatients with stable COPD GOLD grades 2–3. The main differences between the three clinical phenotypes were based on the level of adaptation to the disease as reflected by the relative balance between physiological parameters (severity of airflow limitation) on one hand and the severity of symptoms, functional impairment and quality of life on the other. Over a 1-year period in treatment as usual, no improvement in any subdomain of health status was found. In contrast, after PR, statistically significant and clinically relevant improvements were found in all subdomains of HS, except for airflow limitation. Moreover, the three clinical phenotypes showed a different pattern of change in the subdomains of integral HS with respect to response to PR.

Two phenotypes were identified that were characterized by mild (to moderate) problems in the domains symptoms, functional impairment and quality of life, which were in balance with the airflow limitation. Patients in these phenotypes were labelled adapted to their disease: phenotype 1 (moderate COPD–mild impact on integral HS) and phenotype 3 (severe COPD–mild/moderate impact on integral HS). In contrast, patients of phenotype 2 (moderate COPD–high impact on integral HS) experienced significantly more problems in the domains symptoms, functional impairment and quality of life when compared to the other two phenotypes, despite of having comparable or even less airflow limitation. In these patients, there is an imbalance between disease severity and severity of the other domains of integral HS. Therefore, this phenotype was labelled non-adapted. The present study confirms that adapted and non-adapted patients as described by Effing et al. [14], Nici et al. [15] and Vercoulen [16] can be identified by cluster analyses.

In searching for more homogeneous COPD subgroups, this is the first study that includes such a large set of parameters measuring symptoms, functional impairment and quality of life, in addition to parameters measuring physiological functioning. In line with other studies using cluster analysis [3, 4], we confirmed that identifying subgroups of patients is not based on airflow limitation but on all aspects of health status. In addition, discriminant analysis revealed that all four main domains of integral HS are relevant in this respect, as shown by the fact that one or two subdomains of each main domain were necessary to assign new patients to one of the identified clinical phenotypes.

### Methodological Considerations

In the present study, the vast majority of the study sample were patients with stable, moderate to severe COPD (GOLD grades 2–3). This selection may limit generalizability of results. Ideally, the cluster analysis should be replicated in a group of COPD patients with the complete spectrum of GOLD grades 1–4 to examine whether all possible clinical phenotypes based on integral HS were identified.

In the analyses, we did not include comorbidities as a separate parameter and only a limited set of parameters measuring systemic effects. However, it is unknown whether comorbidities and systemic effects play a role in the capability to adapt. Future studies should examine the role of comorbidities and systemic effects of COPD in these phenotypes.

Sixty-one percent of phenotype 2 report having rheumatoid arthritis, which seems quite high. We doubt that these patients really have rheumatoid arthritis diagnosed by a doctor. These data are based on self-report of patients. Patients in phenotype 2 typically report more symptoms than patients in other phenotypes. Therefore, we interpret these findings as over-reporting by patients.

Due to the low number of patients in phenotype 1 ‘moderate COPD–mild impact on integral HS’ in the PR group, the treatment effect of rehabilitation in phenotype 1 patients should be interpreted with caution.

### Clinical and Research Implications

The findings of the present study and other studies that performed cluster analysis have important implications for clinical practice and future research. These studies have shown that COPD patients represent a very heterogeneous group of persons. Cluster analyses enable identification of more homogeneous subgroups that have been shown to respond differently to pharmacological and non-pharmacological treatment. The present study showed that besides phenotypes based on different (patho)physiological mechanisms, also clinical phenotypes exist that are more determined by behavioural aspects. Knowing to which (patho)physiological phenotype and to which clinical phenotype a patient belongs will enhance personalized treatment by guiding pharmacological and non-pharmacological treatment.

It is well known that a significant number of COPD patients have depression and/or anxiety, and one might assume significant overlap between these patients and the ‘non-adaptive’ COPD patients. In the present study, depression was measured as part of the NCSI subdomain quality of life. We found in 14% of the outpatients and 32% of patients included for rehabilitation a score that indicates depressive symptoms. Anxiety was also measured in the outpatient group and was present in 15% of patients. Although anxiety and depression primarily were found in the non-adapted group, 40% of these non-adapted outpatients did not have depressive symptoms and 59% did not have anxiety. Thus, although clinicians should be aware of the presence of depression and anxiety, this awareness does not capture all patients who are not adapted well to their disease and consequently experience more symptoms, more subjective impairments and worse quality of life as compared to patients with comparable lung function.

Studies to date using cluster analysis to identify different phenotypes recommend to evaluate treatment response [3, 5, 26–29] to identify differences in response between these phenotypes. Although such studies have been performed evaluating the effect of pharmacological treatment [30, 31], similar studies with respect to non-pharmacological treatments are lacking. The present study shows that the identified clinical phenotypes respond differently to TAU as compared to PR. In all three phenotypes, TAU did not show improvements in any subdomain of health status. After PR, statistically and clinically significant improvement was found in all phenotypes for all subdomains, except FEV_1_. The main difference between TAU and PR is that TAU in the Netherlands mainly is characterized by prescribing medication and improving physical fitness and that PR in the present study in addition has a strong focus on teaching the patient self-management skills. From this perspective, it is not surprising that the improvements in health status after PR were most pronounced in the non-adapted patients.

The fact that in the TAU group 20% of patients were classified as non-adapters calls for the need of regular screening of the degree of adaptation to the disease in this group. These patients would profit from interventions aimed at behaviour change in order to improve the patient’s self-management in its many aspects [14, 17].

Phenotype 3 (severe COPD and mild/moderate impaired integral HS) patients are a group of patients that need special attention. Although this group is adapted to the disease, disease severity is high and therefore needs attention. Are these patients super-adapted or are they under-reporting so that problems will emerge at a later moment. In both situations, these patients may be at risk to become non-adapted over time and therefore should be labelled as at risk instead of adapted. From this perspective, regular check-ups of these patients are warranted, because these patients probably cope well with changes and may not seek medical attention unless really needed.

It may seem remarkable that the adapted patients (phenotype 1 and phenotype 3) showed improvements in 4 and 6 of 11 subdomains, respectively. This may suggest that even adapted patients or patients at risk profit from a pulmonary rehabilitation program in addition to TAU, where no improvements were found in any subdomain. The pulmonary rehabilitation program in the present study was a highly intensive multidisciplinary intervention involving eight different disciplines and with a strong focus on increasing adaptation by teaching the patient adequate self-management behaviours. Perhaps in these patients, the same effects might have been found after a less intensive program. Future studies should further investigate the effects of different treatments/modules on the clinical phenotypes and long-term effects. Ideally, this will lead to different interventions for each (clinical) phenotype that guides in choosing the best intervention based on the individual’s needs and preferences at that moment. This will not only help in guiding patient-tailored treatment and improve outcome but also may render treatment to be more cost-effective.

## Conclusions

Three clinical phenotypes were identified by cluster analysis based on the balance or imbalance between physiological functioning on one hand and experienced symptoms, functional impairment and quality of life on the other, reflecting adaptation to the disease. Differences were found between these three clinical phenotypes in TAU and in response to PR. As expected, especially the non-adapted group benefits the most from PR with its emphasis on self-management interventions that enhance adaptation. Identification of subtypes and knowledge of differences in treatment response and differences in development in natural course will help in guiding and optimizing integral patient-tailored treatment, both pharmacological and non-pharmacological*.*

## Appendix

**Table 5:** Change in health status after 1 year in outpatients, for total group and each identified phenotype

	All outpatients (*N* = 143)			Phenotype 1 (*n* = 51) moderate COPD–mild impact health status			Phenotype 2 (*n* = 31) moderate COPD–high impact health status			Phenotype 3 (*n* = 61) severe COPD–mild/moderate impact health status		
	Baseline	12 Months	*p*	Baseline	12 Months	*p*	Baseline	12 Months	*p*	Baseline	12 Months	*p*
Physiological functioning												
FEV_1_% of predicted	52.0 ± 13.8	53.5 ± 14.0	0.06	60.8 ± 11.5	61.2 ± 11.1	0.77	53.3 ± 12.8	53.3 ± 12.5	0.99	44.7 ± 11.7	47.6 ± 13.9	**<0.01**
BMI	25.7 ± 3.9	25.9 ± 4.1	0.19	24.9 ± 3.1	25.0 ± 3.3	0.35	27.1 ± 4.4	27.6 ± 4.8	0.05	25.7 ± 4.1	25.7 ± 4.1	0.96
Symptoms												
Subjective symptoms	7.1 ± 4.7	7.2 ± 4.5	0.94	4.5 ± 3.4	4.9 ± 3.8	0.47	11.4 ± 3.7	10.4 ± 3.5	0.23	7.1 ± 4.6	7.3 ± 4.4	0.65
Dyspnoea emotions	9.7 ± 3.3	10.0 ± 3.3	0.29	9.4 ± 3.2	9.1 ± 3.1	0.62	11.8 ± 3.7	11.7 ± 3.8	0.87	9.0 ± 2.7	9.9 ± 2.9	0.02
Fatigue	26.8 ± 11.0	29.7 ± 11.4	**<0.01**	20.9 ± 7.5	25.8 ± 9.1	**<0.01**	39.6 ± 8.9	40.3 ± 9.6	0.75	25.0 ± 9.1	27.5 ± 10.8	0.04
Functional impairment												
Subjective impairment	9.9 ± 5.0	10.3 ± 4.9	0.16	7.0 ± 2.9	8.0 ± 3.3	0.02	15.8 ± 5.0	14.5 ± 4.4	0.09	9.2 ± 3.8	10.3 ± 4.8	0.13
Behavioural impairment	15.2 ± 13.1	15.2 ± 14.7	0.96	8.0 ± 8.0	7.9 ± 8.4	0.91	30.2 ± 12.4	29.4 ± 16.6	0.80	13.3 ± 10.6	13.9 ± 12.8	0.59
Quality of life												
General QoL	14.0 ± 12.0	15.1 ± 12.7	0.13	9.4 ± 6.0	10.5 ± 8.5	0.34	29.5 ± 13.8	28.5 ± 15.6	0.54	10.0 ± 7.6	12.0 ± 9.0	0.03
HrQoL	4.0 ± 1.6	4.3 ± 1.7	0.09	3.2 ± 0.9	4.0 ± 1.6	**<0.01**	6.0 ± 1.6	5.5 ± 1.3	0.07	3.7 ± 1.3	3.9 ± 1.8	0.39
Satisfaction relations	3.1 ± 1.4	2.9 ± 1.5	0.18	2.8 ± 1.1	2.8 ± 1.6	0.80	4.4 ± 1.9	3.7 ± 1.6	0.02	2.7 ± 0.9	2.7 ± 1.3	0.93

Data are represented as mean ± SD, *p* values in bold: significantly different between baseline and after 1 year (paired *t* test, *p* < 0.01). Data on exercise capacity were not collected after 1 year

*FEV*_*1*_ *%* forced expiratory volume in 1 s, *BMI* body mass index, *VO*_*2*_ *max* maximal oxygen uptake, *QoL* quality of life, *HrQoL* health-related quality of life

**Table 6:** Change in health status after an inpatient pulmonary rehabilitation, for total group and each clinical phenotype

	All patients (*N* = 459)			Phenotype 1 (*n* = 27) moderate COPD–mild impact health status			Phenotype 2 (*n* = 271) moderate COPD–high impact health status			Phenotype 3 (*n* = 161) severe COPD–mild impact health status		
	Before	End	*p*	Before	End	*p*	Before	End	*p*	Before	End	*p*
Physiological functioning												
FEV_1_% of pred	42.8 ± 17.9	43.9 ± 20.6	0.03	60.0 ± 18.6	63.7 ± 20.1	0.22	46.0 ± 18.3	47.2 ± 21.3	0.10	34.2 ± 12.1	34.8 ± 14.6	0.37
BMI	25.9 ± 5.4	25.1 ± 6.6	**<0.01**	23.8 ± 3.8	24.0 ± 3.5	0.28	26.5 ± 6.0	25.3 ± 7.4	**<0.01**	25.2 ± 4.3	24.8 ± 5.6	0.29
VO_2_% of pred^a^	60.8 ± 19.0	64.9 ± 19.3	**<0.01**	92.7 ± 12.0	90.1 ± 17.8	0.53	63.9 ± 16.0	67.7 ± 17.7	**0.01**	50.0 ± 14.0	55.8 ± 15.5	**<0.01**
Symptoms												
Subjective symptoms	13.0 ± 3.8	9.8 ± 4.3	**<0.01**	11.1 ± 3.9	7.7 ± 3.5	**0.01**	13.7 ± 3.6	10.4 ± 4.2	**<0.01**	12.2 ± 4.0	9.1 ± 4.4	**<0.01**
Dyspnoea emotions	12.9 ± 4.1	10.9 ± 4.0	**<0.01**	10.5 ± 2.9	9.8 ± 3.9	0.29	13.9 ± 3.9	11.7 ± 4.1	**<0.01**	11.6 ± 4.2	9.7 ± 3.6	**<0.01**
Fatigue	41.8 ± 9.4	31.5 ± 10.6	**<0.01**	37.9 ± 9.7	28.9 ± 9.2	**<0.01**	45.7 ± 7.3	34.0 ± 9.9	**<0.01**	35.9 ± 9.3	27.8 ± 10.7	**<0.01**
Functional impairment												
Subjective impairment	16.5 ± 5.2	13.0 ± 5.2	**<0.01**	13.3 ± 5.6	9.7 ± 4.1	**<0.01**	17.7 ± 4.9	13.9 ± 5.0	**<0.01**	14.9 ± 5.2	11.9 ± 5.1	**<0.01**
Behavioural impairment	27.4 ± 14.2	25.3 ± 14.5	**<0.01**	11.8 ± 6.4	11.9 ± 9.4	0.95	32.4 ± 14.3	28.4 ± 14.6	**<0.01**	21.4 ± 10.5	22.4 ± 13.2	0.35
Quality of life												
General QoL	26.8 ± 14.8	19.5 ± 12.5	**<0.01**	13.4 ± 7.1	12.3 ± 11.5	0.64	34.3 ± 13.6	22.9 ± 13.0	**<0.01**	16.5 ± 8.6	14.9 ± 9.6	0.05
HrQoL	5.8 ± 1.7	4.1 ± 1.7	**<0.01**	4.7 ± 1.5	3.4 ± 1.8	**0.01**	6.4 ± 1.5	4.3 ± 1.7	**<0.01**	5.1 ± 1.5	3.8 ± 1.6	**<0.01**
Satisfaction relations	3.9 ± 1.8	3.3 ± 1.7	**<0.01**	3.4 ± 1.9	3.1 ± 1.7	0.56	4.3 ± 1.9	3.5 ± 1.7	**<0.01**	3.1 ± 1.5	3.1 ± 1.6	0.82

Data are represented as mean ± SD, *p* values in bold: significantly different before and end rehabilitation (paired *t* test, *p* < 0.01

*FEV*_*1*_ *%* forced expiratory volume in 1 s, *BMI* body mass index, *VO*_*2*_ *max* maximal oxygen uptake, *QoL* quality of life, *HrQoL* health-related quality of life

^a^One hundred fifty-one patients performed a maximal cycle ergometry test at the end of the rehabilitation (respectively, 13/77/61 patients per phenotype); the other patients performed an endurance cycle ergometry (data not shown)

**Table 4:** Discriminant function coefficients for assigning new patients to the clusters

	Phenotype 1 moderate COPD–mild impact HS	Phenotype 2 moderate COPD–high impact HS	Phenotype 3 severe COPD–mild/moderate impact HS
FEV_1_% of predicted	0.379	0.426	0.315
VO_2_% of predicted	0.665	0.546	0.455
Quality of life	0.247	0.446	0.211
Behavioural impairment	0.254	0.408	0.270
Fatigue	0.408	0.578	0.410
Constant	−49.129	−55.470	−29.578
